# Multi-omics analysis of *Populus simonii × P. nigra* leaves under *Hyphantria cunea* stress

**DOI:** 10.3389/fpls.2024.1392433

**Published:** 2024-07-10

**Authors:** Jiahui Jiang, Gaofeng Fan, Ruiqi Wang, Wenjing Yao, Boru Zhou, Tingbo Jiang

**Affiliations:** ^1^ State Key Laboratory of Tree Genetics and Breeding, Northeast Forestry University, Harbin, China; ^2^ Bamboo Research Institute, Nanjing Forestry University, Nanjing, China

**Keywords:** poplar, *Hyphantria cunea*, transcriptome, proteome, metabolome

## Abstract

Poplar is an important greening and timber tree species in China, which has great economic and ecological values. However, the spread of *Hyphantria cunea* has become increasingly serious in recent years, resulting in huge economic loss of poplar production. Exploring the molecular mechanism of poplar reponse to *H. cunea* stress has significant implications for future development of new insect-resistant poplar varieties using genetic engineering technology. In this study, a total of 1039 differentially expressed genes (DEGs), 106 differentially expressed proteins (DEPs) and 212 differentially expressed metabolites (DEMs) were screened from *Populus simonii × P. nigra* leaves under *H. cunea* stress by transcriptome, proteomics and metabolomics analysis, respectively. GO and KEGG analysis showed that the DEGs and DEPs are associated with endopeptidase inhibitor activity, stress response, α-linolenic acid metabolism, phenylpropanoid biosynthesis and metabolic pathways, cysteine and methionine metabolism pathways and MAKP signaling pathway. Metabolomics analysis showed the most of DEMs were lipids and lipid molecules, and the pathways associated with transcriptome mainly include plant hormone signal transduction, α-linolenic acid metabolic pathway, amino sugar and nucleotide sugar metabolism, and phenylpropanoid biosynthesis. In particular, multi-omics analysis showed that several pathways such as α-linolenic acid metabolic, phenylpropanoid biosynthesis and metabolic pathway and cysteine and methionine metabolic pathway were significantly enriched in the three omics, which may play an important role in the resistance to pests in poplar.

## 1 Introduction

*Hyphantria cunea*, a leaf-feeding pest species, is notorious for its extreme adaptability, rapid spread, and ability to inflict severe damage to its host. Therefore, *H. cunea* is difficult to manage and poses a significant threat to agricultural and forestry plants. (Yamanaka et al., 2008; Tang et al., 2012; Wu et al., 2019). In 2021, the cumulative disaster area in China reached 10,427,300 acres, marking an increase of 6.15% compared to 2020, incurring a significant economic losses in the country (Diagne et al., 2021; Ning et al., 2021). Breeding and popularizing new varieties of insect resistant plants is one of the most economical and effective ways to control *H. cunea* epidemic (Sala et al., 2000; Yang et al., 2016).

In the process of plant evolution, a series of defense systems have been established in response to pest stress, including structural barriers, defense compounds and attraction of natural enemies (Howe and Jander, 2008; Agrawal, 2011; Karban, 2011). Trichoid, cuticle and thorn are the first physical barrier layer of plant defense against pests (Hanley et al., 2007). Once the insects break through the physical barrier, plants respond to pest attack by rapidly producing some defense compounds, including insect-resistant proteins, primary and secondary metabolites and plant hormones, etc. (Duffey and Stout, 1996; Abood and Lösel, 2003). Primary metabolites play an important role in plant response to biotic stress (Abood and Lösel, 2003). As the main primary metabolites of plants, amino acids are not only the basis of protein synthesis and the precursor of secondary metabolites, but also the necessary nutrients for the development and reproduction of plant-feeding insects, which are involved in insect defenses in plants (Solomon et al., 2003; Jobic et al., 2007). Secondary metabolites also play an important role in plant adaptation to environmental stresses (Hartmann, 2007; War et al., 2012). Plants phenolics are one of the most common and widely used defense compounds, with strong insecticidal and antibacterial activities (Sharma et al., 2009; War et al., 2012). Among them, lignin is a phenolic heteropolymer that plays a central role in plant defense against insects and pathogens (Johnson et al., 2009). The genes related to lignin development (CAD/CAD-like genes) and synthesis were significantly expressed in plants infested by pests (Barakat et al., 2010). Protease inhibitors (PIs), including cysteine, serine, aspartate and metalloproteinase inhibitors, are essential in plant defenses against pest stress (Ryan, 1990; Lawrence and Koundal, 2002). In detail, PIs can bind and inhibit the activity of digestive enzymes in the gut of plant-eating insects, reducing the digestion of proteins, which leads to insect starvation and even death (Azzouz et al., 2005). The expression level of PIs is low in plants under normal condition, while it is obviously increased after insect infestation or mechanical damage (Divekar et al., 2023). Kunitz trypsin inhibitor (KTI) is a typical serine PI, which is one of the most obvious defense proteins in response to insect feeding. So far, a total of 22 KTI genes have been identified in poplar, whose gene expression level and protein abundance are significantly increased under pest injury (Major and Constabel, 2008).

In addition, many hormone-mediated signal transduction pathways participate in plant response to pest stress (Verhage et al., 2010). For example, Jasmonic acid (JA) is a key plant hormone in plant response to insect defense, which is derived from linolenic acid via octadecane pathway (Shivaji et al., 2010). When plants are subjected to pest feeding, JA content accumulates rapidly, and many genes related to pest defense are regulated by JA (Pauwels et al., 2009; Shivaji et al., 2010). For instance, JA can affect plant growth by inhibiting the activity of calcium-dependent protein kinases (CDPK) in potato (Ulloa et al., 2002). Salicylic acid (SA) is a simple phenolic compound produced in plants, which is considered as a new type of plant growth regulator in inducing disease and abiotic stress resistances (Rivas-San Vicente and Plasencia, 2011). More specifically, it can activate the defense mechanism by enhancing the stability of cell wall and improving the antioxidant capacity. Moreover, SA can kill a variety of pests (Zhao et al., 2009). Abscisic acid (ABA) is also closely related to plant defense regulation (Zhang and Klessig, 2001). For example, protein phosphatase 2C (PP2C) is a negative ABA signal regulator. Plants with mutation of the genes encoding PP2C exhibit ABA hypersensitivity. Overexpression of *ZmPP2C* in Arabidopsis resulted in reduced tolerance to osmotic stress (Liu et al., 2009). In addition, plant resistance to feeding stress is also related to oxidation state (Felton et al., 1994; Torres, 2010). The antioxidant enzymes such as peroxidase (POD), lipoxygenase (LOX) and phenylalanine transferase (PAL) can prevent the absorption and utilization of nutrients by insects (Maffei et al., 2007; Bruinsma et al., 2009). Under aphid stress, POD activity of aphid resistant wheat was 3 times higher than that of susceptible varieties (He et al., 2011).

As an important timber and greening tree species in the world, poplar has great economic and ecological values. In recent years, the production of poplar has been severely affected by *H. cunea* stress (Wu et al., 2019; Ning et al., 2021). Therefore, *H. cunea* has become one of the important limiting factors for the sustainable development of poplar plantation in China (Ning et al., 2021). In this study, the differentially expressed genes (DEGs), differential expressed proteins (DEPs) and differential expressed metabolites (DEMs) of *Populus simonii × P. nigra* in response to *H. cunea* stress were revealed through multi-omics analysis. KEGG enrichment analysis was used to analyze the regulatory patterns of key pathway genes and metabolites. Based on the results, a molecular mechanism of pest stress response was constructed in poplar. The fundamental resources provided by this study will be helpful in identification of key pest resistance genes and genetic engineering efforts aimed at breeding of new insect-resistant poplar varieties.

## 2 Materials and methods

### 2.1 Plant materials

In this study, the tissue culture plants of *Populus simonii × P. nigra* were maintained in the biological clean room of Northeast Forestry University. The 20-day-old sterile seedlings were transferred to greenhouse for soil cultivation. After 30 days, the 72 seedlings with similar growth state were divided into 12 groups and those of 6 groups were selected for insect feeding. We conducted insect attack treatment by placing five third instar larvae of *H. cunea* on the leaves of poplar plants in mesh bag. After 48 hours, the damaged leaves of the treated plants and the leaves at the same position of the control plants were collected and stored at -80°C, respectively. The plant samples were transported with dry ice to Novogene (China), in which four biological replicates were used for RNA sequencing and proteome analysis, and six biological replicates for metabolome analysis.

### 2.2 Transcriptome sequencing

The high-throughput output resulted in a large amount of high-quality raw data with scores of Q30 or higher for most bases. The low-quality reads, such as those containing connectors, were completely removed to obtain clean reads. And the resulting clean reads were mapped to *Populus trichocarpa* genome using HISAT 2 software (https://phytozome-next.jgi.doe.gov/). The mapped reads were then assembled and quantified using String Tie. The FPKM values of the genes were calculated using RSEM.

### 2.3 Quantitative proteome analysis

Quantitative proteome was performed using Tandem Mass Tags (TMT) technology as follows: proteins were extracted according to the previous method (Rao et al., 2021). Protein concentrations were assayed using BCA experimental kit. The peptides were solubilized with 0.5 M TEAB and labelled with the TMT kit (Thermo Fisher Scientific, USA). Liquid chromatography-electrospray ionization-tandem mass spectrometry (LC-ESI-TMS) analysis was then performed as described in the literature (Li et al., 2017). Based on *Populus trichocarpa* genome database, the raw files obtained from mass spectrometry detection were compared to identify proteins. Peptide, protein and parent ion mass tolerance distribution analyses were performed to assess the quality of the mass spectrometry data. Proteome Discoverer software was used for database searching and quantification of spectral peptide and protein.

### 2.4 Metabolome profiling

Metabolites were analyzed based on high resolution mass spectrometry (HRMS) detection as follows: the samples were processed and detected according to the previous methods (Barri and Dragsted, 2013). The scan range was selected as m/z 100–1500. Offline raw data were analyzed using CD3.3 software. The data were firstly screened with parameters such as retention time and mass-to-charge ratio. The data were then compared with the secondary spectral databases mzCloud and mzVault and the primary database Mass List to identify metabolites. Metabolites with a coefficient of variance (CV) of less than 30% in the QC samples were retained as final identifications for subsequent analysis.

### 2.5 The screening of DEGs, DEPs and DEMs

For DEGs, |log2(FC)|>1 and P-value<0.05 were used as thresholds. For DEPs, FC>1.2 or FC<0.833 and P-value<0.05 were used as thresholds. For DEMs, VIP>1.0, FC>1.2 or FC<0.833 and P-value<0.05 were used as thresholds. For metabolites, VIP>1.0, FC>1.2 or FC<0.833 and P-value<0.05 were used as thresholds. The obtained DEGs, DEPs and DEMs were analyzed for GO and KEGG enrichment respectively. Enrichment analysis and visualization were performed using TBtools software (Chen et al., 2023).

### 2.6 qRT-PCR

qRT-PCR was used to verify the accuracy of RNA-Seq results. Actin was used as the internal control, and three biological replicates were performed for each procedure (Regier and Frey, 2010). All primers used in this study are listed in **Supplementary Table S5**. The normalization of the data was performed according to the instructions for the GoTaq 2-Step qRT-PCR System Kit and by the 2^−ΔΔCt^ method (Livak and Schmittgen, 2001).

### 2.7 Data analysis

Standard errors and standard deviations were calculated by *t test*. Statistically significant level was set to *p*-value ≤ 0.05. The data were presented as mean ± standard error (SE) with each SE being calculated from four or six biological repeats..

## 3 Results

### 3.1 Transcriptome analysis of poplar in response to pest stress

**Figure 1:**
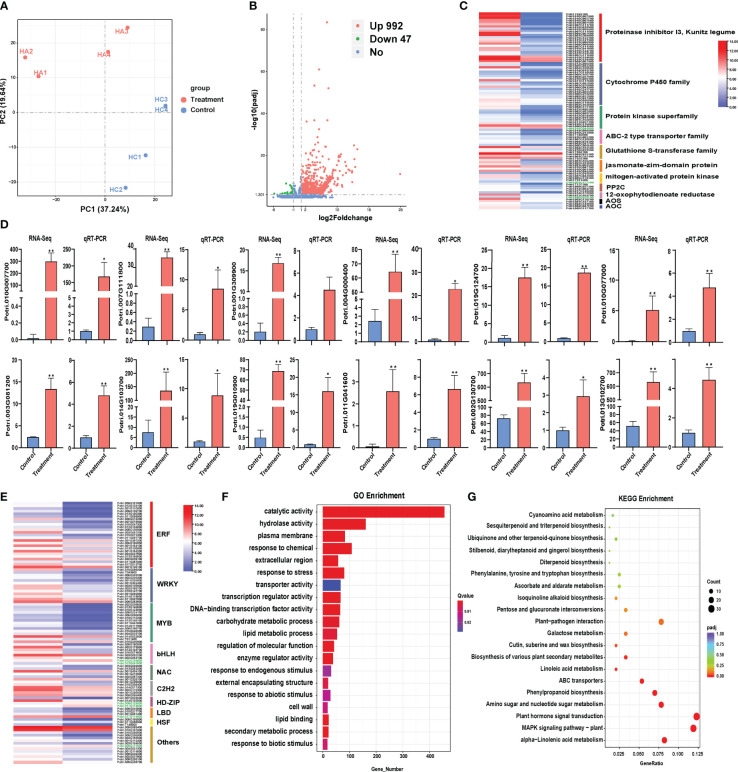
Transcriptome analysis of Populus simonii × P. nigra under H. cunea stress. **(A)** PCA of transcriptome sequencing. **(B)** Identification of differentially expressed genes (DEGs). UP represents up-regulated genes and DOWN represents down-regulated genes. **(C)** qRT-PCR verification of RNA-Seq results. Treatment and Control samples were with and without insect treatment, respectively. The error bars indicate the standard deviations of the biological replicates. “*” indicates P < 0.05 between Treatment and Control samples; “**” indicates P < 0.01. **(D)** Classification of major functional genes in DEGs. **(E)** Classification of transcription factors in DEGs. **(F)** The Top 20 GO enrichment analysis of DEGs. **(G)** The top 20 KEGG enrichment analysis of DEGs.

The differentially expressed genes (DEGs) of *Populus simonii × P. nigra* under *H. cunea* stress were analyzed by RNA-Seq. A total of 47.71 Gb high-quality clean data was generated from 8 sequencing libraries, with an average of 5.96 Gb per library. There were 86.18% of the reads mapped to the genome of *Populus trichocarpa*. Among them, the reads with score of Q30 accounted for 93.91%, and the average GC content was 43.94% (**Supplementary Table S1**). Principal component analysis (PCA) showed that the eight samples were clustered into two groups: treatment group and control group (**Figure 1A**).

There were a total of 1039 DEGs in poplar under the *H. cunea* stress of which 992 DEGs were up-regulated and 47 were down-regulated (**Figure 1B**). To validate the accuracy of RNA-Seq, 12 DEGs were randomly selected to detect their relative expression level by qRT-PCR. The results showed that the expression trends of the 12 DEGs were consistent in RNA-Seq analysis and qRT-PCR results, indicating that the transcriptome sequencing data were reliable (**Figure 1D**). Cluster analysis showed that treatment group and control group were clustered into two different categories, and the gene expression pattern was similar within each category (**Supplementary Figure 1**).

There were 98 transcription factor (TF) genes and 82 functional genes related to pest stress identified in *Populus simonii × P. nigra* transcriptome (**Supplementary Table S2**). The functional genes related to adversity stress mainly include protease inhibitors (23), cytochrome P450 superfamily proteins (20), protein kinase superfamily proteins (11), ABC transporter family proteins (7), glutathione S transferases (7) and JA hormone-related genes (14) (**Figure 1C**). The 98 TFs contain ERF, WRKY, MYB, bHLH, C2H2, NAC and other TF family genes. Among them, ERF (25), WRKY (13), MYB (15), and bHLH (8) accounted for the majority (**Figure 1E**).

Based on GO enrichment analysis, the 1039 DEGs were significantly enriched into 146 GO terms, including 93 terms associated with biological processes, 48 molecular functions and 5 cellular components. Significant enrichment terms were involved in catalytic activity, plasma membrane, response to chemical and response to stress. In addition, multiple GO terms were identified to be related to plant stress resistance, including biotic stimulus, abiotic stimulus, transcription regulator activity, lipid metabolic process and enzyme regulator activity (**Figure 1F**). KEGG pathway enrichment analysis showed that many pathways related to plant stress resistance were significantly enriched, including α-linolenic acid metabolism, MAPK hormone signal transduction, plant hormone signal transduction, amino sugar and nucleotide sugar metabolism, phenylpropanoid biosynthesis, ABC transporter, linoleic acid metabolism pathway, glutathione metabolism and plant pathogen interaction (**Figure 1G**).

### 3.2 Proteomic analysis of poplar in response to insect stress

**Figure 2:**
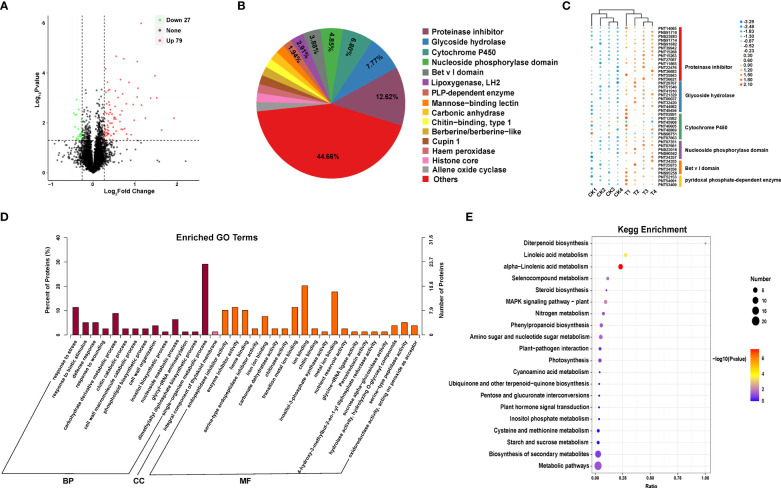
Proteomic analysis of Populus simonii × P. nigra under H. cunea stress. **(A)** Identification of differentially expressed proteins (DEPs). UP represents up-regulated proteins and DOWN represents downregulated proteins. **(B)** Domain analysis of DEPs. **(C)** Cluster analysis of major DEPs. **(D)** GO enrichment analysis of DEPs. **(E)** The top 20 KEGG enrichment analysis of DEPs.

TMT-labeled quantitative proteomics was used to analyze the differentially expressed proteins of poplar under pest stress. A total of 7094 proteins were detected, and as many as 106 differentially expressed proteins (DEPs) were identified. Among them, 79 DEPs were up-regulated and 27 were down-regulated (**Figure 2A**).

Protein domain analysis showed that protease inhibitors, glycoside hydrolase, cytochrome P450 and nucleoside phosphorylase accounted for 12.62%, 7.77%, 6.80% and 4.85%, respectively, which were the four kinds of proteins with the highest frequency (**Figures 2B, C**). GO enrichment and KEGG functional annotation showed that these DEPs were significantly enriched into 35 GO terms (p ≤ 0.05), including 14 terms associated with biological processes, 20 molecular functions and 1 cellular component. Among the 14 terms associated with biological processes, the terms involved in response to stress, carbohydrate derivative metabolic process, nucleoside metabolic process and single metabolic organism process, which had the highest enrichment abundance. In addition, the terms associated with defense response and biotic stimulus were also significantly enriched. Among the 20 terms associated with molecular functions, the terms of ion binding, metal ion binding, enzyme inhibitor activity, endopeptidase inhibitor activity and transition metal ion binding were the most abundant. The one terms associated with cellular component was enriched in integral components of thylakoid membrane (**Figure 2D**). KEGG enrichment analysis revealed a total of 24 significant enrichment pathways, including α-linolenic acid metabolism, phenylpropanoid biosynthesis pathway, amino sugar and nucleotide sugar metabolism, and MAPK signaling pathway (**Figure 2E**).

### 3.3 Association analysis of transcriptome and proteome of poplar in response to pest stress

**Figure 3:**
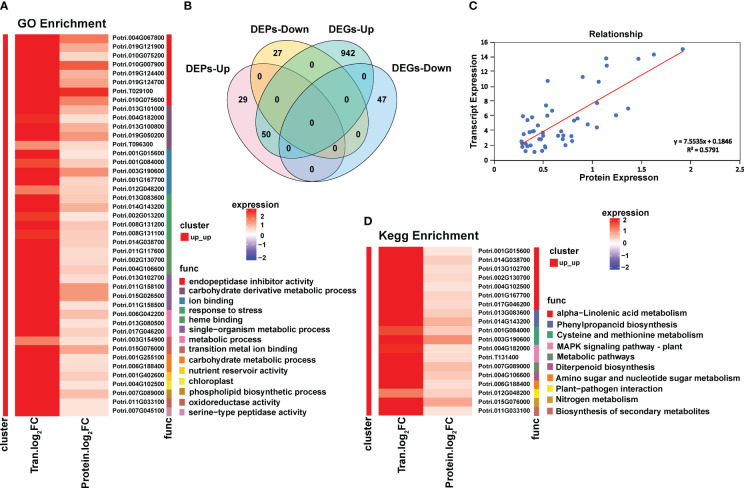
Combined analysis of transcriptome and proteome of Populus simonii × P. nigra under H. cunea stress. **(A)** GO enrichment analysis of co-upregulated DEGs and DEPs. **(B)** Wayne diagram of coupregulated DEGs and DEPs. **(C)** Correlation analysis of co-upregulated DEGs and DEPs. **(D)** KEGG enrichment analysis of co-upregulated DEGs and DEPs.

The 106 DEPs accounted for 10.2% of the 1039 DEGs. The correlation between the expression level of DEPs and the corresponding gene expression level was analyzed. There were 50 DEPs displaying similar expression trends with their corresponding genes, both of which were significantly up-regulated (**Figure 3B**). The correlation coefficient between the two was 0.5791 (**Figure 3C**).

Among the 50 DEPs, there were 8 trypsin inhibitor family proteins, 7 phosphorylase superfamily proteins, 4 proteins involved in the α-linolenic acid pathway, and 3 cytochrome P450 family proteins identified. In particular, there was an EF-hand protein with a MYB domain (**Supplementary Table S3**).

GO enrichment results showed that the significantly enriched terms included endopeptidase inhibitor activity, carbohydrate derivative metabolic process, ion binding, stress response, heme binding and single organism metabolism (**Figure 3A**). KEGG enrichment found 10 significantly enriched metabolic pathways, including α-linolenic acid metabolic pathway, phenylpropanoid biosynthesis pathway, cysteine and methionine metabolic pathway and MAKP signal transduction pathway. The α-linolenic acid metabolic pathway was significantly enriched at both transcriptome and proteome level (**Figure 3D**).

### 3.4 Metabolomics analysis of poplar in response to pest stress

**Figure 4:**
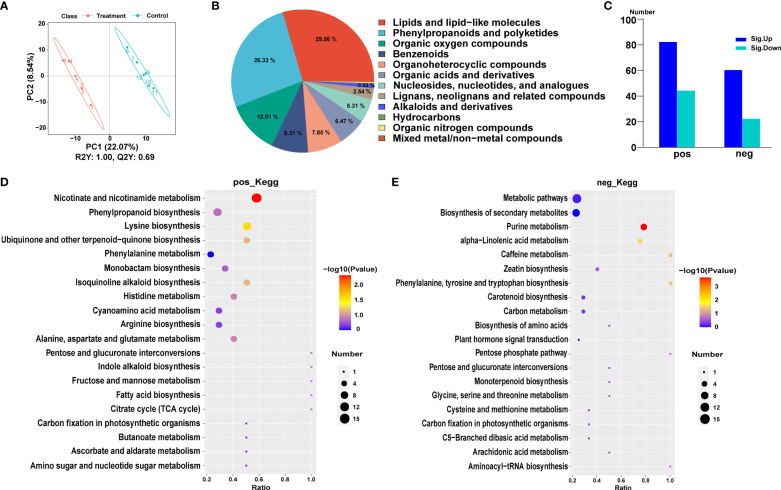
Metabolic analysis of Populus simonii × P. nigra under H. cunea stress. **(A)** PCA of metabolome sequencing. **(B)** Classification of differentially expressed metabolites (DEMs). **(C)** Identification of DEMs. POS represents cationic metabolites and NEG represents anionic metabolites. UP represents up-regulated metabolites and DOWN represents down-regulated metabolites. **(D)** The top 20 KEGG enrichment analysis of cationic DEMs. **(E)** The top 20 KEGG enrichment analysis of anion DEMs.

Differentially expressed metabolites (DEMs) of *Populus simonii × P. nigra* under pest stress were identified by liquid chromatography-mass spectrometry (LC-MS). Partial least squares discriminant analysis (PLS-DA) was used to distinguish the difference among the different treatments and different replicates. The variation between different treatments (PC1) and the variation between different replicates (PC2) accounted for 22.07% and 8.54% of the total variation, respectively (**Figure 4A**). With the standards of VIP > 1.0, FC > 1.2 or FC < 0.833 and P-value < 0.05, a total of 212 DEMs were identified, including 128 cationic DEMs, of which 83 DEMs were up-regulated and 45 were down-regulated. There were 84 anion DEMs including 61 up-regulated DEMs and 23 down-regulated DEMs (**Figure 4C**). And the DEMs can be divided into 12 categories, including lipid and lipid molecules, phenylpropanoids and polyketides, organic oxygenates and various free amino acids (**Figure 4B**).

The enriched cationic DEMs can be clustered into 36 metabolic pathways, including nicotinic acid and nicotinamide metabolic pathways, lysine biosynthesis metabolism, phenylpropanoid biosynthesis pathway, quinoline alkaloid biosynthesis, ubiquinone and other terpenoid-quinone biosynthesis pathways, and phenylalanine metabolic pathway (**Figure 4D**). The enriched anion DEMs were classified in 21 metabolic pathways, including purine metabolic pathway, α-linolenic acid metabolism, caffeine metabolism and phenylalanine, tyrosine and tryptophan biosynthesis pathways (**Figure 4E**). Both anion and cation DEMs were related to amino acid biosynthesis and energy metabolism pathways, such as the biosynthesis of cysteine, methionine, arginine, histidine, alanine, aspartic acid, and glutamic acid. At the same time, the DEMs associated with TCA cycle and glucose metabolism pathway also changed significantly.

**Figure 5:**
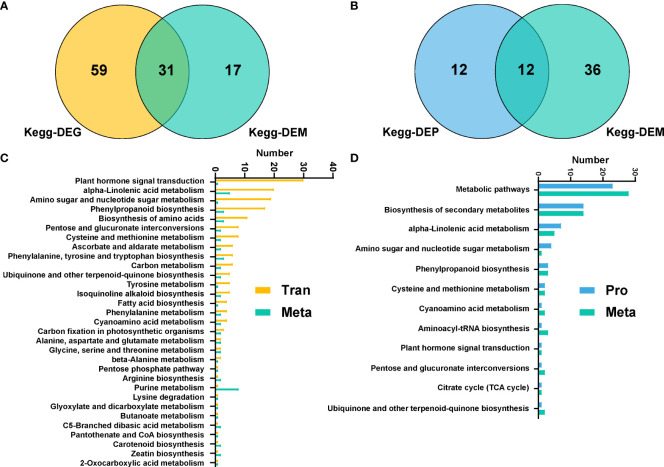
Multi-omics analysis of Populus simonii × P. nigra under H. cunea stress. **(A)** Wayne diagram of KEGG pathways in transcriptome and metabolome. **(B)** Venn diagram of KEGG pathways of proteome and metabolome. **(C)** Quantitative analysis of co-enriched DEGs and DEMs in KEGG pathways. **(D)** Quantitative analysis of co-enriched DEPs and DEMs in KEGG pathways.

Association analysis of transcriptome and metabolome showed that a total of 31 metabolic pathways were significantly enriched in DEGs and DEMs, including plant hormone signal transduction, α-linolenic acid metabolic pathway, amino sugar and nucleotide sugar metabolism, phenylpropanoid biosynthesis and other pathways (**Figures 5A, C**). And association analysis of proteomics and metabolomics revealed that there were 12 pathways enriched in DEPs and DEMs simultaneously, including metabolic pathways, biosynthesis of secondary metabolites, α-linolenic acid metabolism, etc. (**Figures 5B, D**).

### 3.5 Regulatory pathways of poplar in response to pest stress

**Figure 6:**
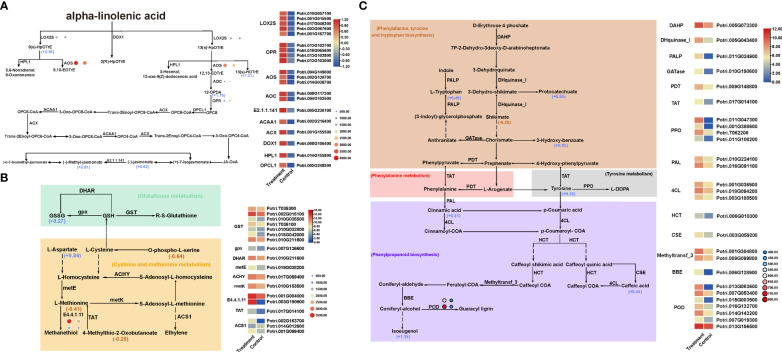
Co-enriched KEGG pathways in transcriptome, proteome and metabolome. **(A)** α-linolenic acid pathway. **(B)** Glutathione metabolism, Cysteine and methionine metabolism pathways. **(C)** Phenylalanine, tyrosine and tryptophan biosynthesis, phenylalanine metabolism, and phenylpropanoid biosynthesis pathways. The rectangles in the figure represent DEGs; the circles represent DEPs and the size of the circle represents their expression level; the number next to the metabolite represents log2FC value.

Multi-omics analysis showed that the DEGs, DEPs and DEMs were significantly enriched in α-linolenic acid metabolic pathway (**Figure 6A**), cysteine and methionine metabolism (**Figure 6B**) and phenylpropanoid biosynthesis and metabolic pathway (**Figure 6C**), etc..

Among them, α-linolenic acid metabolism (**Figure 6A**) is an important way to produce JA, which plays a key role in plant insect resistance. Transcriptome sequencing revealed there were 20 DEGs closely related to α-linolenic acid metabolic pathway, all of which were up-regulated, encoding LOX2S, AOC, OPR, AOS, E2.1.1.141, ACAA1, ACX, DOX1, HPL1, OPCL1 and other 10 enzyme proteins, which were key enzymes in the α-linolenic acid metabolic pathway. The expression trends of 6 DEGs encoding LOX2S, AOC, OPR and AOS were consistent with those of 4 DEPs. In addition, five DEMs including 13(s) -HOTrE, 9(s)-HpOTrE, 12-OPDA, JA and MeJA were closely related to α-linolenic acid metabolic pathway. Among them, 9(s)-HpOTrE synthesis was closely related to the transcription and expression of *LOX2S* gene. 12-OPDA synthesis was closely related to the transcription and expression of two genes encoding oxidene cyclase (AOC). JA O-methyltransferase (E2.1.1.141) was a key enzyme in the conversion efficiency of JA to methyl jasmonate (MeJA) (**Figure 6A**).

Phenylpropanoid compounds are not only an important part of woods, but also widely involved in the process of insect resistance, disease resistance, UV radiation resistance and anti-aging in woody plants. RNA-seq revealed a total of 27 DEGs encoding 14 enzymes were involved in the biosynthesis and metabolism of phenylalanine, tyrosine and tryptophan, and 7 out of them encoded peroxidase (POD). Moreover, the DEGs encoding amidase (TAT) and prephenate dehydratase (PDT) in the phenylalanine metabolic pathway, the DEGs encoding polyphenol oxidase (PPO) in the tyrosine metabolic pathway, and the DEGs encoding tryptophan synthase (PALP) in the tryptophan synthesis pathway were all up-regulated. In addition, two POD DEPs were up-regulated (**Figure 6C**). Metabolomic analysis showed that the 7 DEMs including tyrosine, tryptophan, cinnamic acid, Caffeic acid, Isoeugenol, Protocatechuate and 3-Hydroxy-benzoate were up-regulated. Among them, the up-regulated expression of cinnamic acid, caffeic acid and isoeugenol in the metabolic group was significantly correlated with the up-regulated expression of the genes involved in phenylalanine ammonia lyase (PAL) and caffeoyl shikimate esterase (CSE) (**Figure 6C**). It shows that the biosynthesis and metabolic pathway of phenylpropanoid compounds is an important way in poplar under *H. cunea* stress.

Glutathione is a tripeptide containing γ-amide bond and sulfhydryl group, which is composed of glutamic acid, cysteine and glycine. And it has antioxidant effect in plant stress resistance. Transcriptome sequencing revealed that there were 8 DEGs encoding GST, DHAR and gpx in the glutathione metabolic pathway, of which 6 DEGs encoded glutathione S-transferase (**Figure 6B**). Correspondingly, the expression of glutathione sulfide GSSG was significantly up-regulated in the metabolome. In addition, there were 9 DEGs encoding 6 enzymes, including metE, ACHY, metK, E4.4.1.11, TAT and ACS1, which were related to cysteine and methionine metabolic pathways. Among them, three DEGs encoded aminocyclopropane-1-carboxylic acid synthase (ACS1), two of them were up-regulated and one (Potri.014G012600) was down-regulated. The DEGs encoding methionine-γ-lyase (E4.4.1.11) were significantly up-regulated, which showed same expression trend in the proteome, promoting methionine degradation. Metabolomics analysis showed that a total of four DEMs were related to cysteine and methionine metabolic pathways, including Methionine, L-Aspartate, 4-Methylthio-2-Oxobutanoate and O-phospho-L-serine, of which only L-Aspartate was up-regulated. And the down-regulated expression of 4-Methylthio-2-Oxobutanoate was related to the expression change of TAT genes (**Figure 6B**).

## 4 Discussion

As the main lepidopteran pest of woody plants, *H. cunea* seriously restricts the sustainable development of poplar plantations, which causes significant economic losses (Moon et al., 2021). It is of great significance to excavate the endogenous insect-resistant genes for cultivating insect-resistant poplar varieties and preventing the environmental pollution caused by chemical control in the future. Plants rapidly conduct signal transduction through kinase cascades, reactive oxygen species, plant hormones and other signaling pathways under pest stress (Müller et al., 2019). In this study, we conducted multi-omics analysis of *Populus simonii* × *P. nigra* under pest stress. The DEGs, DEPs and DEMs related to JA synthesis, phenylalanine, tyrosine, tryptophan, glutathione and methionine metabolic pathways were identified to display significant changes, indicating that these pathways play an important role in response to pest stress in poplar.

### 4.1 Jasmonic acid and its derivatives produced by the α-linolenic acid pathway under *H.cunea* stress

Plant tissue damage caused by caterpillars and beetles can activate JA-based signal transduction, which induces JA (Kandoth et al., 2007). JA precursors and JA derivatives play a key role in mediating plant response to pests (Xu et al., 2021). α-Linolenic acid is one of the precursors of JA biosynthesis, which can be converted into 12-oxo phytodienoic acid (12-OPDA) by lipoxygenase (LOX), allene oxide synthase (AOS) and allene oxide cyclase (AOC). And 12-OPDA can be converted into JA under the action of peroxisome-localized OPDA reductase (OPR), which can further derive MeJA (Wasternack and Feussner, 2018). The expression levels of JA biosynthesis genes, *LmLOX2* and *LmAOS* were significantly increased in lupin seeds with MeJA treatment. In addition, a few JA -related defense genes such as *LmTPS1*, *LmTPS4*, *LmPI2*, *LmMBL*, *LmLODC*, *LmCSD1*, and *LmPOD* were also induced (Erazo-Garcia et al., 2021). In this study, the α-linolenic acid metabolic pathway was significantly enriched in the transcriptome, proteome and metabolome in *Populus simonii × P. nigra* under *H. cunea* stress. At transcriptome and proteome levels, the DEGs and DEPs encoding *LOX*, *AOS*, *AOC*, etc. were up-regulated in poplar plants. At the same time, the up-regulated DEMs detected in the metabolome included JA, MeJA and 12-OPDA, as well as JA derivatives such as propyl jasmonate and jasmone. In particular, the DEGs related to the transport of OPDA from chloroplasts to peroxisomes is up-regulated in the transcriptome of poplar under pest stress. It indicated that *OPR* genes play an important role in the biosynthesis of 12-OPDA, which was key enzymes in the metabolic pathway of α-linolenic acid and JA synthesis.

Studies have shown that MeJA can induce the production of protease inhibitors (PIs). Treatment of *C.medinalis* and MeJA in rice main stem significantly increased the level of PIs in the leaves of rice at early tillering stage (Tong et al., 2023). MeJA can also induce the accumulation of PI I and PI II in rice leaves (Farmer et al., 1992). In this study, we found that the DEGs encoding PIs was up-regulated by pest stress, which was the highest frequency in all DEGs, accounting for 2.2%. At the same time, the DEPs containing PI domain accounted for 12.62% in all DEPs, which was the highest frequency in all DEPs categories. GO enrichment analysis also indicated PIs were significantly enriched in poplar under pest stress. It shows that pest stress can induce the expression of PIs genes through JA pathway to resist pest feeding in poplar.

### 4.2 Glycoside conjugates and sulfur content increased in plants under *H. cunea* stress

Previous study proved that glycoside-bound volatiles and various flavonoids were significantly increased in tea plant under green leafhopper stress, and the related DEGs encoding the above secondary metabolites were significantly up-regulated, while the glutathione content was significantly reduced (Zhao et al., 2020). Glutathione sulfide is an important oxidation product of glutathione after stress in plants (Dixon et al., 2005). In this study, the DEGs encoding glutathione peroxidase (gpx) and deoxyascorbate reductase (DHAR) were significantly up-regulated in poplar under pest stress, and the concentration of glutathione sulfide increased significantly. The contents of amino acids, alkaloids and phenols in the leaves of passion fruit were significantly increased after infection of *A. pallida* (Yang et al., 2023). Aspartic acid (Asp) is not only a component of protein, but also a downstream metabolite necessary for regulating plant growth and resisting environmental stress (Han et al., 2021). The content of Asp increased significantly when the tomato seedlings were infected by nematodes, indicating that Asp plays an important role in the response to nematode stress (Bali et al., 2020). In this study, metabolomics analysis showed that the content of Asp in poplar increased significantly under the stress of *H. cunea*. Through the combined analysis of transcriptome and metabolome, it was further found that amino acid metabolic pathways related to Asp were enriched, such as cysteine and methionine metabolism. The results of metabolomics also showed that the content of methionine and O-phospho-L-serine decreased in the metabolic pathway of cysteine and methionine. Among them, O-phospho-L-serine is the precursor of methionine, which participates in threonine pathway. And methionine can be converted into glucosinolates, which can accumulate nearly 30% of the total sulfur content in plant organs, displaying a repellent effect on pests (Hirai et al., 2007). In this study, the DEGs and DEPs encoding Methionine- γ- Lyase *E4.4.1.11* were significantly up-regulated in poplar under pest stress. The results indicated that methionine was decomposed under the action of *E4.4.1.11*, which was conducive to further synthesis of glucosinolates, improving the resistance of plants to pests.

### 4.3 Biosynthesis and metabolism of aromatic amino acids under *H.cunea* stress

Aromatic amino acids (phenylalanine, tyrosine and tryptophan) in plants are not only essential components of protein synthesis (Maeda and Dudareva, 2012), but also precursors of various secondary metabolites. The secondary metabolites produced by aromatic amino acids are mainly phenylpropanoids, and their biosynthesis is initiated by the activity of phenylalanine ammonia lyase (PAL) (Tzin and Galili, 2010). In general, phenylpropanoid metabolism takes a small number of intermediates in the shikimic acid pathway as the core, producing a large number of secondary metabolites (Vogt, 2010). They are not only representative indicators of plant stress response to light or mineral treatment, but also key medium for pest resistance in plants. The expression of phenylalanine metabolic genes such as *PAL* and 4-coumarate-CoA ligase (*4CL*) can be induced by tea green leafhopper, as well as some genes encoding *POD* and shikimate-O-hydroxycinnamoyl transferase (*HCT*) in the phenylpropanoid pathway (Camera et al., 2004). In this study, two genes encoding PAL, one gene encoding HCT, three genes encoding 4CL, and seven genes encoding POD were up-regulated. And the proteins encoding POD were also up-regulated, indicating that these two genes play a more important role in pest response in poplar. Phenylpropanoid is traditionally synthesized by phenylalanine, however, the second synthetic method of phenylpropanoid is through tyrosine synthesis in several plants (Beaudoin-Eagan and Thorpe, 1985). In this study, the genes encoding *PALP* and *TAT* were significantly up-regulated, which promoted the accumulation of tryptophan and tyrosine. Studies have shown that *MYB165* can inhibit the synthesis or accumulation of flavonoids in poplar suspension cells. Overexpression of *MYB165* in transgenic poplars reduces the expression of Potri.011G024900, which was a key gene for β-chain of tryptophan synthase (Ma et al., 2018). In our study, one DEG (Potri.011G024900) encoding *PALP* was up-regulated in poplar under pest stress, and the accumulation of tryptophan was increased, indicating that the gene plays an important role in the synthesis and accumulation of tryptophan.

### 4.4 Lipid metabolites under *H.cunea* stress

Lipids are main components of cell membranes, and some lipid molecules play an important role in signal transduction of plants under biotic and abiotic stresses (Xie et al., 2023). In this study, a large of DEMs were identified as lipids, accounting for 29.56% of all DEMs, which include lipid metabolites such as lysophospholipid (LPL) and cucurbitacin. LPL is a lipid-derived metabolic intermediate in the cell membrane, which regulates many important biological processes as an important biologically active signaling molecule (Cao et al., 2023). Previous studies have shown that LPL may be involved in the signal transduction of stress response, and its expression is induced by salt and osmotic stresses (Meijer et al., 2001). The accumulation of a variety of LPL may be more conducive to rapid transmission of stress signals in plants (Arisz and Munnik, 2011). Cucurbitacin (Cuc) is a highly oxidized triterpenoid compound, which is widely found in cucurbitaceae plants and some other plant families, which plays an important role in plant resistance to pathogens and pests. The bitterness produced by Cuc-B in melon is considered to be a defensive response to repel pests and herbivores (Dong et al., 2021). The addition of Cuc-B or Cuc-E to the diet of *Drosophila larvae* can inhibit its peeling and even cause its death (Toyofuku et al., 2021). Cuc-I, Cuc-B and Cuc-E all affect the feeding behavior of beetles on cucumber and zucchini (Yang et al., 2005). In this study, a variety of LPL, Cuc-B, Cuc-I and Cuc-E were induced to express in poplar under pest stress, which may enhance stress signal transduction and induce the repellent to pests, thereby improving pest resistance in poplar.

## 5 Conclusion

In this study, the transcriptome, proteome and metabolomics of *Populus simonii* × *P. nigra* leaves under *H. cunea* stress were profiled, resulting in 1039 DEGs, 106 DEPs and 212 DEMs, respectively. The DEGs involved 146 GO terms, including 93 biological processes, 48 molecular functions and 5 cellular components. DEPs were significantly enriched into 35 GO terms, including 14 biological processes, 20 molecular functions and 1 cellular component. DEMs were significantly enriched to 57 metabolic pathways. Among them, DEGs, DEPs and DEMs were all significantly enriched in the α-linolenic acid metabolic pathway, phenylalanine biosynthesis and metabolic pathway, cysteine metabolic pathway and methionine metabolic pathway. The final products of these metabolic pathways include protease inhibitors, JA and its derivatives, aspartate, tyrosine and tryptophan (**Supplementary Table S4**), which are important substances for pest resistance in poplar.

## Data availability statement

All data generated or analyzed during this study are included in this published article and information files. The RNA-sequencing data NCBI’s SRA with the accession number PRJNA1067315. The mass spectrometry proteomics data have been deposited to the ProteomeXchange Consortium (https://proteomecentral.proteomexchange.org) via the iProX partner repository with the dataset identifier PXD048856. The metabolome data used during this study have been deposited in the MetaboLights, which has been assigned the unique identifier MTBLS9940 (https://www.ebi.ac.uk/metabolights/ MTBLS9940).

## Author contributions

JJ: Data curation, Formal analysis, Software, Writing – original draft, Writing – review & editing. GF: Data curation, Formal analysis, Writing – original draft. RW: Data curation, Writing – review & editing. WY: Writing – review & editing. BZ: Writing – review & editing. TJ: Funding acquisition, Writing – review & editing.

## References

[B1] Abood J. K. Lösel D. M. (2003). Changes in carbohydrate composition of cucumber leaves during the development of powdery mildew infection. Plant Pathol. 52, 256–265. doi: 10.1046/j.1365-3059.2003.00814.x

[B2] Agrawal A. A. (2011). Current trends in the evolutionary ecology of plant defence. Funct. Ecol. 25, 420–432. doi: 10.1111/j.1365-2435.2010.01796.x

[B3] Arisz S. A. Munnik T. (2011). The salt stress-induced LPA response in Chlamydomonas is produced via PLA2 hydrolysis of DGK-generated phosphatidic acid. J. Lipid Res. 52, 2012–2020. doi: 10.1194/jlr.M016873 21900174 PMC3196233

[B4] Azzouz H. Cherqui A. Campan E. D. M. Rahbé Y. Duport G. Jouanin L.. (2005). Effects of plant protease inhibitors, oryzacystatin I and soybean Bowman–Birk inhibitor, on the aphid Macrosiphum euphorbiae (Homoptera, Aphididae) and its parasitoid Aphelinus abdominalis (Hymenoptera, Aphelinidae). J. Insect Physiol. 51, 75–86. doi: 10.1016/j.jinsphys.2004.11.010 15686649

[B5] Bali S. Kaur P. Jamwal V. L. Gandhi S. G. Sharma A. Ohri P.. (2020). Seed priming with jasmonic acid counteracts root knot nematode infection in tomato by modulating the activity and expression of antioxidative enzymes. Biomolecules 10, 98. doi: 10.3390/biom10010098 31936090 PMC7022828

[B6] Barakat A. Bagniewska-Zadworna A. Frost C. J.. (2010). Phylogeny and expression profiling of CAD and CAD-like genes in hybrid Populus (P. deltoides× P. nigra): evidence from herbivore damage for subfunctionalization and functional divergence. BMC Plant Biol. 10, 1–11. doi: 10.1186/1471-2229-10-100 20509918 PMC2887455

[B7] Barri T. Dragsted L. O. (2013). UPLC-ESI-QTOF/MS and multivariate data analysis for blood plasma and serum metabolomics: effect of experimental artefacts and anticoagulant. Anal. Chim. Acta 768, 118–128. doi: 10.1016/j.aca.2013.01.015 23473258

[B8] Beaudoin-Eagan L. D. Thorpe T. A. (1985). Tyrosine and phenylalanine ammonia lyase activities during shoot initiation in tobacco callus cultures. Plant Physiol. 78, 438–441. doi: 10.1104/pp.78.3.438 16664262 PMC1064755

[B9] Bruinsma M. Posthumus M. A. Mumm R. Mueller M. J. van Loon J. J. A. Dicke M.. (2009). Jasmonic acid-induced volatiles of Brassica oleracea attract parasitoids: effects of time and dose, and comparison with induction by herbivores. J. Exp. Bot. 60, 2575–2587. doi: 10.1093/jxb/erp101 19451186 PMC2692006

[B10] Camera S. L. Gouzerh G. Dhondt S. Hoffmann L. Fritig B. Legrand M.. (2004). Metabolic reprogramming in plant innate immunity: the contributions of phenylpropanoid and oxylipin pathways. Immunol. Rev. 198, 267–284. doi: 10.1111/j.0105-2896.2004.0129.x 15199968

[B11] Cao X. van Putten J. P. M. Wösten M. M. S. M. (2023). Biological functions of bacterial lysophospholipids. Adv. Microb. Physiol. 82, 129–154. doi: 10.1016/bs.ampbs.2022.10.001 36948653

[B12] Chen C. Wu Y. Li J. Wang X. Zeng Z. Xu J.. (2023). TBtools-II: A "one for all, all for one" bioinformatics platform for biological big-data mining. Mol. Plant 16, 1733–1742. doi: 10.1016/j.molp.2023.09.010 37740491

[B13] Diagne C. Leroy B. Vaissière A. C. Gozlan R. E. Roiz D. Jarić I.. (2021). High and rising economic costs of biological invasions worldwide. Nature 592, 571–576. doi: 10.1038/s41586-021-03405-6 33790468

[B14] Divekar P. A. Rani V. Majumder S. Karkute S. G. Molla K. A. Pandey K. K.. (2023). Protease inhibitors: an induced plant defense mechanism against herbivores. J. Plant Growth Regul. 42, 6057–6073. doi: 10.1007/s00344-022-10767-2

[B15] Dixon D. P. Skipsey M. Grundy N. M. Edwards R. J. (2005). Stress-induced protein S-glutathionylation in arabidopsis. Plant Physiol. 138, 2233–2244. doi: 10.1104/pp.104.058917 16055689 PMC1183410

[B16] Dong L. Almeida A. Pollier J. Khakimov B. Bassard J. E. Miettinen K.. (2021). An independent evolutionary origin for insect deterrent cucurbitacins in Iberis amara. Mol. Biol. Evol. 38, 4659–4673. doi: 10.1093/molbev/msab213 34264303 PMC8557398

[B17] Duffey S. S. Stout M. J. (1996). Antinutritive and toxic components of plant defense against insects. Arch. Insect Biochem. 32, 3–37. doi: 10.1002/(SICI)1520-6327(1996)32:1

[B18] Erazo-Garcia M. P. Sotelo-Proaño A. R. Ramirez-Villacis D. X. Garcés-Carrera S. Leon-Reyes A. J. (2021). Methyl jasmonate-induced resistance to Delia platura (D iptera: A nthomyiidae) in Lupinus mutabilis. Pest Manag. Sci. 77, 5382–5395. doi: 10.1002/ps.6578 34313385

[B19] Farmer E. E. Johnson R. R. Ryan C. (1992). Regulation of expression of proteinase inhibitor genes by methyl jasmonate and jasmonic acid. Plant Physiol. 98, 995–1002. doi: 10.1104/pp.98.3.995 16668777 PMC1080300

[B20] Felton G. Summers C. Mueller A. J. (1994). Oxidative responses in soybean foliage to herbivory by bean leaf beetle and three-cornered alfalfa hopper. J. Chem. Ecol. 20, 639–650. doi: 10.1007/BF02059604 24242118

[B21] Han M. Zhang C. Suglo P. Sun S. Wang M. Su T. (2021). L-Aspartate: An essential metabolite for plant growth and stress acclimation. Molecules 26, 1887. doi: 10.3390/molecules26071887 33810495 PMC8037285

[B22] Hanley M. E. Lamont B. B. Fairbanks M. M. Rafferty C. M. (2007). Plant structural traits and their role in anti-herbivore defence. Perspect. Plant Ecol. 8, 157–178. doi: 10.1016/j.ppees.2007.01.001

[B23] Hartmann T. J. (2007). From waste products to ecochemicals: fifty years research of plant secondary metabolism. Phytochemistry 68, 2831–2846. doi: 10.1016/j.phytochem.2007.09.017 17980895

[B24] He J. Chen F. Chen S. Lv G. Deng Y. Fang W.. (2011). Chrysanthemum leaf epidermal surface morphology and antioxidant and defense enzyme activity in response to aphid infestation. J. Plant Physiol. 68, 687–693. doi: 10.1016/j.jplph.2010.10.009 21145126

[B25] Hirai M. Y. Sugiyama, Sawada K. Tohge Y. Obayashi T. Suzuki T.. (2007). Omics-based identification of Arabidopsis Myb transcription factors regulating aliphatic glucosinolate biosynthesis. Proc. Natl. Acad. Sci. U S A. 104, 6478–6483. doi: 10.1073/pnas.0611629104 17420480 PMC1849962

[B26] Howe G. A. Jander G. J. (2008). Plant immunity to insect herbivores. Annu. Rev. Plant Biol. 59, 41–66. doi: 10.1146/annurev.arplant.59.032607.092825 18031220

[B27] Jobic C. Boisson A. M. Gout E. Rascle C. Fèvre M. Cotton P.. (2007). Metabolic processes and carbon nutrient exchanges between host and pathogen sustain the disease development during sunflower infection by Sclerotinia sclerotiorum. Planta 226, 251–265. doi: 10.1007/s00425-006-0470-2 17219185

[B28] Johnson M. T. Smith S. D. Rausher M. D. (2009). Plant sex and the evolution of plant defenses against herbivores. Proc. Natl. Acad. Sci. U S A. 106, 18079–18084. doi: 10.1073/pnas.0904695106 19617572 PMC2775293

[B29] Kandoth P. K. Ranf S. Pancholi S. S. Jayanty S. Walla M. D. Miller W.. (2007). Tomato MAPKs LeMPK1, LeMPK2, and LeMPK3 function in the systemin-mediated defense response against herbivorous insects. Proc. Natl. Acad. Sci. U S A. 104, 12205–12210. doi: 10.1073/pnas.0700344104 17623784 PMC1924534

[B30] Karban R. J. (2011). The ecology and evolution of induced resistance against herbivores. Funct. Ecol. 25, 339–347. doi: 10.1111/j.1365-2435.2010.01789.x

[B31] Lawrence P. K. Koundal K. R. (2002). Plant protease inhibitors in control of phytophagous insects. ELECTRON. J. BIOTECHN. 5, 5–6. doi: 10.2225/vol5-issue1-fulltext-3

[B32] Li J. Ren L. Gao Z. Jiang M. Liu Y. Zhou L.. (2017). Combined transcriptomic and proteomic analysis constructs a new model for light-induced anthocyanin biosynthesis in eggplant (Solanum melongena L.). Plant Cell Environ. 40, 3069–3087. doi: 10.1111/pce.13074 28940206

[B33] Liu L. Hu,. X. Song J. Zong X. Li D. Li D. J. (2009). Over-expression of a Zea mays L. protein phosphatase 2C gene (ZmPP2C) in Arabidopsis thaliana decreases tolerance to salt and drought. J. Plant Physiol. 166, 531–542. doi: 10.1016/j.jplph.2008.07.008 18930563

[B34] Livak K. J. Schmittgen T. D. (2001). Analysis of relative gene expression data using real-time quantitative PCR and the 2(-Delta Delta C(T)) Method. Methods 25, 402–408. doi: 10.1006/meth.2001.1262 11846609

[B35] Ma D. Reichelt M. Yoshida K. Gershenzon J. Constabel C. P. (2018). Two R2R3-MYB proteins are broad repressors of flavonoid and phenylpropanoid metabolism in poplar. Plant J. 96, 949–965. doi: 10.1111/tpj.14081 30176084

[B36] Maeda H. Dudareva N. J. (2012). The shikimate pathway and aromatic amino acid biosynthesis in plants. Annu. Rev. Plant Biol. 63, 73–105. doi: 10.1146/annurev-arplant-042811-105439 22554242

[B37] Maffei M. E. Mithöfer A. Boland W. J. (2007). Insects feeding on plants: rapid signals and responses preceding the induction of phytochemical release. Phytochemistry 68, 2946–2959. doi: 10.1016/j.phytochem.2007.07.016 17825328

[B38] Major I. T. Constabel C. P. (2008). Functional analysis of the Kunitz trypsin inhibitor family in poplar reveals biochemical diversity and multiplicity in defense against herbivores. Plant Physiol. 146, 888–903. doi: 10.1104/pp.107.106229 18024557 PMC2259082

[B39] Meijer H. J. Arisz S. A. Van Himbergen J. A. Musgrave A. Munnik T. J. (2001). Hyperosmotic stress rapidly generates lyso-phosphatidic acid in Chlamydomonas. Plant J. 25, 541–548. doi: 10.1046/j.1365-313x.2001.00990.x 11309144

[B40] Moon J. H. Won S. J. Maung C. E. H. Choi J. H. Choi S. I. Ajuna H. B.. (2021). The Role of Lysobacter antibioticus HS124 on the control of fall webworm (Hyphantria cunea Drury) and growth promotion of Canadian poplar (Populus canadensis Moench) at Saemangeum reclaimed land in Korea. Microorganisms 9, 1580. doi: 10.3390/microorganisms9081580 34442659 PMC8398145

[B41] Müller N. A. Kersten B. Fladung M. Schroede H. J. (2019). RNA-seq of eight different poplar clones reveals conserved up-regulation of gene expression in response to insect herbivory. BMC Genomics 20, 673. doi: 10.1186/s12864-019-6048-8 31455224 PMC6712675

[B42] Ning J. Lu P. Fan J. Ren L. Zhao L. (2021). American fall webworm in China: A new case of global biological invasions. Innovation (Camb). 3, 100201. doi: 10.1016/j.xinn.2021.100201 35036980 PMC8753265

[B43] Pauwels L. Inzé D. Goossens A. J. (2009). Jasmonate-inducible gene: what does it mean? Trends Plant Sci. 14, 87–91. doi: 10.1016/j.tplants.2008.11.005 19162528

[B44] Rao S. Yu T. Cong X. Lai X. Xiang J. Cao J.. (2021). Transcriptome, proteome, and metabolome reveal the mechanism of tolerance to selenate toxicity in Cardamine violifolia. J. Hazard Mater. 406, 124283. doi: 10.1016/j.jhazmat.2020.124283 33187796

[B45] Regier N. Frey B. (2010). Experimental comparison of relative RT-qPCR quantification approaches for gene expression studies in poplar. BMC Mol. Biol. 11, 57. doi: 10.1186/1471-2199-11-57 20701777 PMC2930637

[B46] Rivas-San Vicente M. Plasencia J. (2011). Salicylic acid beyond defence: its role in plant growth and development. J. Exp. Bot. 62, 3321–3338. doi: 10.1093/jxb/err031 21357767

[B47] Ryan C. A. (1990). Protease inhibitors in plants: genes for improving defenses against insects and pathogens. Annu.rev.phytopathol 28, 425–449. doi: 10.1146/annurev.py.28.090190.002233

[B48] Sala F. Castiglione S. Jianjun H. Zheng Y. Han Y. (2000). “Field and molecular evaluation of insect-resistant transgenic poplar (Populus nigra L.) trees,” in Plant genetic engineering: towards the third millennium: Proceedings of the International Symposium on Plant Genetic Engineering, Havana, Cuba, Vol. 5. 137–142. doi: 10.1016/S0168-7972(00)80021-3

[B49] Sharma H. C. Sujana G. Manohar R. D. (2009). Morphological and chemical components of resistance to pod borer, Helicoverpa armigera in wild relatives of pigeonpea. Arthropod-plant Inte 3, 151–161. doi: 10.1007/s11829-009-9068-5

[B50] Shivaji R. Camas A. Ankala A. Engelberth J. Tumlinson J. H. Williams W. P.. (2010). Plants on constant alert: elevated levels of jasmonic acid and jasmonate-induced transcripts in caterpillar-resistant maize. J. Chem. Ecol. 36, 179–191. doi: 10.1007/s10886-010-9752-z 20148356

[B51] Solomon P. S. Tan K. C. Oliver R. P. (2003). The nutrient supply of pathogenic fungi; a fertile field for study. Mol. Plant Pathol. 4, 203–210. doi: 10.1046/j.1364-3703.2003.00161.x 20569380

[B52] Tang R. Zhang J. P. Zhang Z. N. (2012). Electrophysiological and Behavioral Responses of Male Fall Webworm Moths (Hyphantria cunea) to Herbivory-Induced Mulberry (Morus alba) Leaf Volatiles. PloS One 7, e49256. doi: 10.1371/journal.pone.0049256 23166622 PMC3498160

[B53] Tong L. Wu W. Lin Y. Chen D. Zeng R. Lu L.. (2023). Insect herbivory on main stem enhances induced defense of primary tillers in rice (Oryza sativa L.). Plants (Basel). 12, 1199. doi: 10.3390/plants12051199 36904060 PMC10005496

[B54] Torres M. A. (2010). ROS in biotic interactions. Physiol. Plant 138, 414–429. doi: 10.1111/j.1399-3054.2009.01326.x 20002601

[B55] Toyofuku M. Fujinaga D. Inaba K. Funahashi T. Fujikawa Y. Inoue H.. (2021). The plant-derived triterpenoid, cucurbitacin B, but not cucurbitacin E, inhibits the developmental transition associated with ecdysone biosynthesis in Drosophila melanogaster. J. Insect Physiol. 134, 104294. doi: 10.1016/j.jinsphys.2021.104294 34389412

[B56] Tzin V. Galili G. (2010). The biosynthetic pathways for shikimate and aromatic amino acids in Arabidopsis thaliana. Arabidopsis Book. 8, e0132. doi: 10.1199/tab.0132 22303258 PMC3244902

[B57] Ulloa R. Raíces M. MacIntosh G. Maldonado S. Téllez-Iñón M. (2002). Jasmonic acid affects plant morphology and calcium-dependent protein kinase expression and activity in Solanum tuberosum. Physiol. Plant 115, 417–427. doi: 10.1034/j.1399-3054.2002.1150312.x 12081535

[B58] Verhage A. Van W. Pieterse C. (2010). Plant immunity: it’s the hormones talking, but what do they say? Plant Physiol. 154, 536–540. doi: 10.1104/pp.110.161570 20921180 PMC2949039

[B59] Vogt T. (2010). Phenylpropanoid biosynthesis. Mol. Plant 3, 2–20. doi: 10.1093/mp/ssp106 20035037

[B60] War A. Paulraj M. Ahmad T. Buhroo A. Hussain B. Ignacimuthu S.. (2012). Mechanisms of plant defense against insect herbivores. Plant Signal Behav. 7, 1306–1320. doi: 10.4161/psb.21663 22895106 PMC3493419

[B61] Wasternack C. Feussner I. (2018). The oxylipin pathways: biochemistry and function. Annu. Rev. Plant Biol. 69, 363–386. doi: 10.1146/annurev-arplant-042817-040440 29166128

[B62] Wu N. Zhang S. Li X. Cao Y. Liu X. Wang Q.. (2019). Fall webworm genomes yield insights into rapid adaptation of invasive species. Nat. Ecol. Evol. 3, 105–115. doi: 10.1038/s41559-018-0746-5 30532047

[B63] Xie Q. Liu B. Dong W. Li J. Wang D. Liu Z.. (2023). Comparative transcriptomic and metabolomic analyses provide insights into the responses to NaCl and Cd stress in Tamarix hispida. Sci. Total Environ. 884, 163889. doi: 10.1016/j.scitotenv.2023.163889 37142042

[B64] Xu J. Wang X. Zu H. Zeng X. Baldwin I. T. Lou Y.. (2021). Molecular dissection of rice phytohormone signaling involved in resistance to a piercing-sucking herbivore. New Phytol. 230, 1639–1652. doi: 10.1111/nph.17251 33533489

[B65] Yamanaka T. Tatsuki S. Shimada M. (2008). Adaptation to the new land or effect of global warming? An age-structured model for rapid voltinism change in an alien lepidopteran pest. J. Anim. Ecol. 77, 585–596. doi: 10.1111/j.1365-2656.2008.01367.x 18266693

[B66] Yang M. Li H. Qiao H. Guo K. Xu R. Wei H.. (2023). Integrated Transcriptome and Metabolome Dynamic Analysis of Galls Induced by the Gall Mite Aceria pallida on Lycium barbarum Reveals the Molecular Mechanism Underlying Gall Formation and Development. Int. J. Mol. Sci. 24, 9839. doi: 10.3390/ijms24129839 37372986 PMC10298420

[B67] Yang R. L. Wang A. X. Zhang J. Dong Y. Yang M. S. Wang J. M. (2016). Genetic transformation and expression of transgenic lines of Populus × euramericana with insect-resistance and salt-tolerance genes. Genet. Mol. Res. 15. doi: 10.4238/gmr.15028635 27173305

[B68] Yang X. Kong C. Liang W. Zhang M. Hu F. (2005). Relationships of Aulacophora beetles feeding behavior with cucurbitacin types in host crops. Ying Yong Sheng Tai Xue Bao. 16, 1326–1329. doi: 10.1360/aps040037 16252877

[B69] Zhang S. Klessig D. F. (2001). MAPK cascades in plant defense signaling. Trends Plant Sci. 6, 520–527. doi: 10.1016/s1360-1385(01)02103-3 11701380

[B70] Zhao L. Y. Chen J. L. Cheng D. F. Sun J. R. Liu Y. Tian Z. (2009). Biochemical and molecular characterizations of Sitobion avenae-induced wheat defense responses. Crop Prot. 28, 435–442. doi: 10.1016/j.cropro.2009.01.005

[B71] Zhao X. Chen S. Wang S. Shan W. Wang X. Lin Y.. (2020). Defensive responses of tea plants (Camellia sinensis) against tea green leafhopper attack: a multi-omics study. Front. Plant Sci. 10. doi: 10.3389/fpls.2019.01705 PMC6978701 32010173

