# Supplementary figures and images for: Multi-omics analysis of Populus simonii × P. nigra leaves under Hyphantria cunea stress
Source: Front Plant Sci. 2024 Jul 10;15:1392433. doi: 10.3389/fpls.2024.1392433 (PMC11267504; doi:10.3389/fpls.2024.1392433)

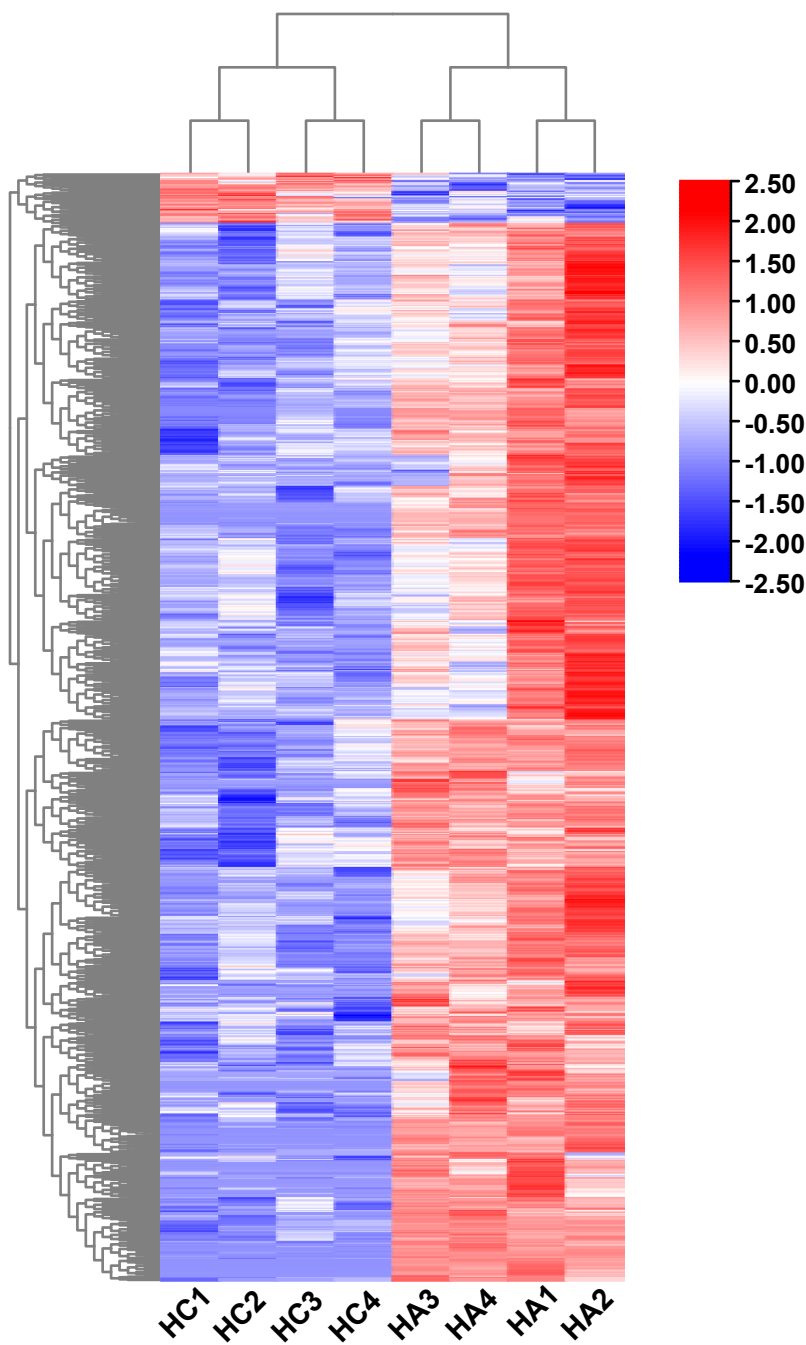

**DEGs clustering heatmap**

Supplement: Supplementary file 1 [file DataSheet_1.pdf]
